# Identification and validation of pyroptosis patterns in AML via comprehensive bioinformatics analysis

**DOI:** 10.1007/s12672-025-02298-5

**Published:** 2025-04-10

**Authors:** Zeyu Deng, Hongkai Zhu, Zhao Cheng, Ruijuan Li, Hongling Peng

**Affiliations:** 1 Department of Hematology, The Second Xiangya Hospital of Central South University, Changsha, Hunan People’s Republic of China; 2 Institute of Hematology, Central South University, Changsha, Hunan People’s Republic of China; 3 Hunan Engineering Research Center of Cell Immunotherapy for Hematopoietic Malignancies, Changsha, Hunan People’s Republic of China; 4 Hunan Key Laboratory of Tumor Models and Individualized Medicine, Changsha, Hunan People’s Republic of China

**Keywords:** Acute myeloid leukemia, Classification, Pyroptosis, Immune, Prognosis

## Abstract

Pyroptosis, a lytic inflammatory cell death mechanism, plays dual roles in tumorigenesis, but its clinical relevance in acute myeloid leukemia (AML) remains poorly understood. Through an integrative analysis of 40 pyroptosis-related genes in newly diagnosed AML patients (TCGA, n = 151) and healthy controls (GTEx, n = 386), we identified 32 genes with aberrant expression. Among these, 9 genes were found to be significant prognostic markers, including ELANE (protective), and CASP1, CHMP4B, BAK1, and CHMP2A (risk), which retained their prognostic significance after adjusting for age and gender. Using unsupervised nonnegative matrix factorization (NMF) on TCGA data, we classified AML into two pyroptosis patterns: the ELANE^high^ subtype, associated with favorable survival, and the ELANE^low^ subtype, which was enriched in poor karyotypes and adverse outcomes. This classification was validated in an independent cohort (GSE10358, n = 91). Single-cell RNA sequencing data (GSE116256, n = 15) revealed that the ELANE^low^ subtype is characterized by an immunologically active microenvironment, marked by an expansion of cytotoxic T cells and naive CD4 + /CD8 + T cells. Factor analysis revealed associations between pyroptosis patterns and other forms of cell death, including ferroptosis, autophagy, and apoptosis, as well as with karyotype, leukemia stemness, and TP53/FLT3-ITD mutations. Prognostic immune gene sets enriched in the ELANE^low^ subtype were associated with interferon signaling and ubiquitin-mediated degradation pathways. Furthermore, protein–protein interaction (PPI) network analysis identified three sub-networks and nine key hub genes. This study integrates gene expression data from newly diagnosed AML patients, revealing the heterogeneity of pyroptosis patterns within the population. It highlights the potential links between distinct pyroptosis patterns, the immune microenvironment, various cell death pathways, leukemia stemness, and genomic alterations, offering novel biomarkers and therapeutic targets for risk stratification and immunomodulatory interventions in AML.

**Supplementary Information:**

The online version contains supplementary material available at 10.1007/s12672-025-02298-5.

## Introduction

Acute myeloid leukemia (AML) is characterized by malignant proliferation of myeloid primordial cells in hematopoietic system. It is a highly heterogeneous disease group, which can be transformed from hematopoietic stem/progenitor cells at different differentiation stages. Chemotherapy and hematopoietic stem cell transplantation are important treatment methods. However, Despite the emergence of new treatment approaches, the five-year survival rate for patients with relapsed and refractory AML remains only 10% [1]. Traditional AML classification relies mainly on conventional markers and leukemia cell morphology, and cannot reflect the different clinical course of individual patients. Therefore, it is very important to stratify AML and seek the associated prognostic gene sets for the accurate treatment of AML.

Multiple factors are closely associated with the development of AML, such as proto-oncogene activation, inflammation, and ferroptosis [2–4]. Pyroptosis is a new type of programmed cell death, which is different from apoptosis and necrosis. It can be triggered by various intracellular or extracellular stimuli. Pyroptosis is mainly mediated by caspase-1/3/4/5/11, inflammasome and gasdermin protein family, and is characterized by cell swelling, membrane dissolution and release of inflammatory factors [5]. Jia et al. reported that compared with AML patients with complete remission, the expression levels of NLR family pyrin domain-containing 3 (NLRP3), apoptosis-related spot like protein (ASC) and interleukin-18 (IL-18) in bone marrow monocytes of newly diagnosed AML patients were significantly increased [6]. In addition, pyroptosis was closely related to the infiltration of immune cells in tumor microenvironment, indicating that it may be a target for immunotherapy [7, 8]. However, the prognostic roles of pyroptosis-related genes and the correlation between pyroptosis and immunity in AML remain to be revealed.

Kecheng Huang et al. [9] used an unsupervised clustering method with pyroptosis-related gene sets to divide AML samples into two groups, C1 and C2. However, the authors focused on constructing a prognostic model based on pyroptosis-related genes, which is similar to the approach taken by Huifang Zhang et al. [10], who used a gene pair method to build a prognostic model. Both studies did not clearly define the differences between the two groups, and the pyroptosis risk groups derived from the prognostic model scores do not correspond to the baseline pyroptosis patterns in AML. In contrast, this study classifies AML samples based on the unbiased expression of pyroptosis-related genes and explores the potential associations between pyroptosis patterns, other cell death modalities, genomic alterations, and leukemia stemness in newly diagnosed AML samples. We mapped the pyroptosis model onto single-cell data using a gene pair random forest model and analyzed the immune microenvironment differences between the two pyroptosis subtypes. Additionally, we applied GSVA to analyze the enrichment differences across numerous immune datasets in the two patterns. Our aim is to uncover the potential molecular and immune mechanisms underlying the pyroptosis patterns in the AML population.

## Methods

### Data collection

The gene expression data of healthy human peripheral blood samples are included in Genotype-Tissue Expression (GTEx) database [11]. The Cancer Genome Atlas (TCGA) database contains acute myeloid leukemia patients' transcriptome and clinical data [12]. UCSC Xena [13] website (https://xena.ucsc.edu/) embodying biological data from GTEx and TCGA was used to download the content above. Another dataset, GSE10358, with gene expression data and survival data of AML, was acquired in Gene Expression Omnibus (GEO) database. Single-cell transcriptome data of GSE116256 was obtained in Gene Expression Omnibus (GEO), which contain 16 AML patients and 5 healthy control (Supplementary Table 1). The AML samples included in this study are all from newly diagnosed patients. 40 pyroptosis related genes and 5219 immunologic genes sets were obtained from Gene Set Enrichment Analysis (GSEA) website (https://www.gsea-msigdb.org/gsea/msigdb/index.jsp) (Supplementary Table 2) [14]. Genes associated with ferroptosis (WP_FERROPTOSIS.v2024.1.Hs.gmt), apoptosis (WP_APOPTOSIS.v2024.1.Hs.gmt), and autophagy (WP_AUTOPHAGY.v2024.1.Hs.gmt) were also downloaded from the GSEA website. Genes related to cuproptosis were retrieved from the literature (Supplementary Table 2) [15, 16].

### Data preprocessing

Gene expression matrix of AML patients in TCGA and healthy samples in GTEx were merged, and get Variance Stabilized Data function of DEseq2 package along with sva package were used to make normalization and remove batch effect. Transcriptome values were made log2 transform in both TCGA and GEO cohorts. As for single-cell data, we applied the “Seurat” package to conduct analysis for single-cell data. UMAP method was used to make Non-linear dimensional reduction.

### GSVA and functional enrichment Analysis

Gene set variation analysis (GSVA) can measure the enrichment extent of specific gene sets corresponding to the biological function upon a sample population [17]. Besides, we performed the Molecular Function (MF) enrichment analysis and Reactome pathway analysis using the clusterProfiler package.

### Statistical analysis

All analyses conducted in our study were executed by R software (version 4.0.5). p < 0.05 was regarded as significant. Mann–Whitney U test was used in differential analysis for pyroptosis-related genes between two groups. Limma package was used to make differential analysis for enrichment score (ES) of gene sets between two clusters. Correlations analysis between the pyroptosis-related genes were evaluated using the Pearson method. Parameter statistical test of cox regression analysis was conducted using the WALD test method. The number of features were determined using Ten-fold cross validation in cox lasso regression. P values were corrected using the False discovery rate (FDR) method. In survival analysis, p values were evaluated by the log-rank test. Nonnegative matrix factorization (NMF) is an effective unsupervised cluster method, which was extensively used to identify cancer molecular subtypes. CancerSubtypes package was utilized to perform NMF analysis and visualization work [18]. Heatmap was fabricated with Pheatmap package, and we made boxplot using ggpubr package.

All methods were performed in accordance with the relevant guidelines and regulations.

## Results

### Expression of pyroptosis-related genes in AML

**Fig. 1:**
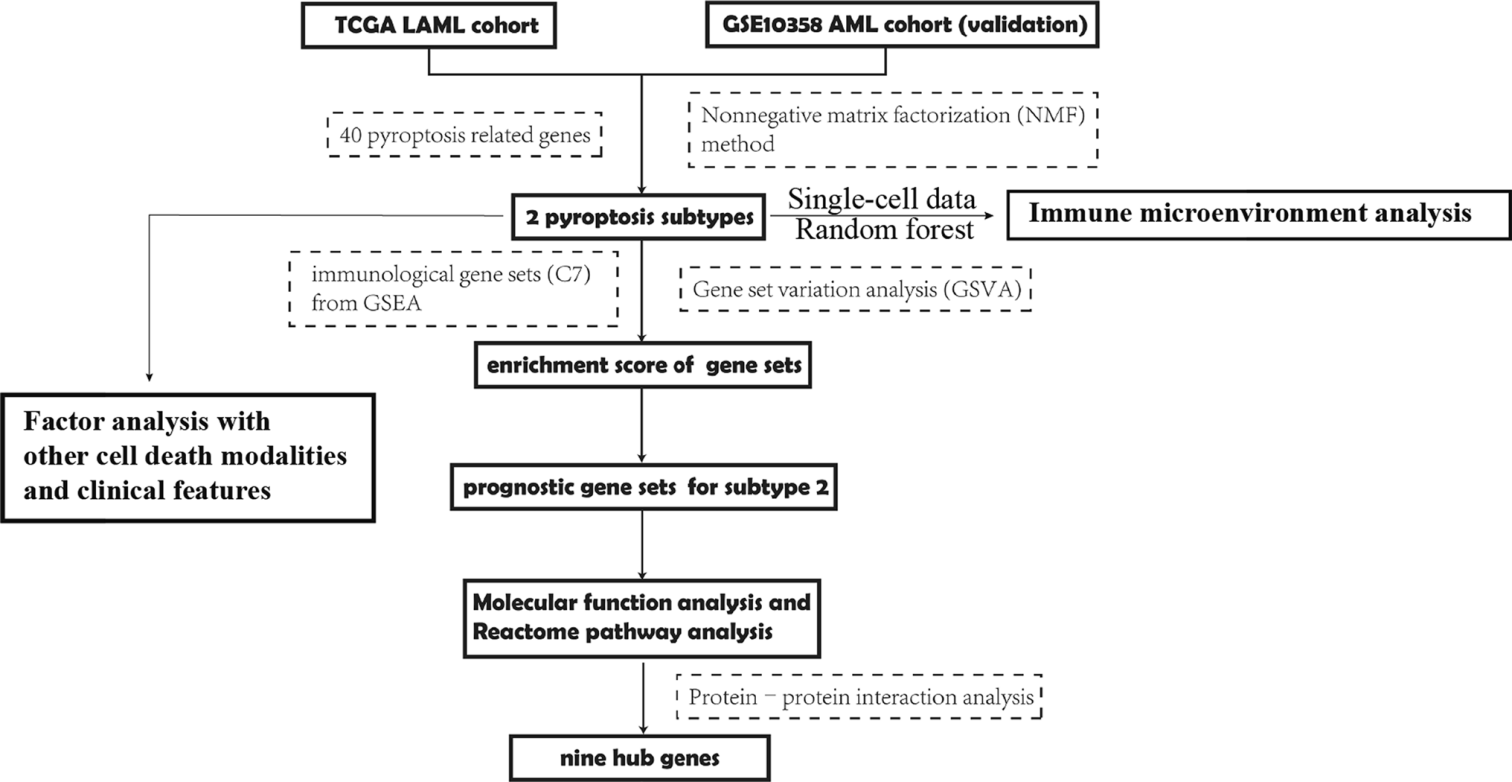
Flowchart of our study

**Fig. 2:**
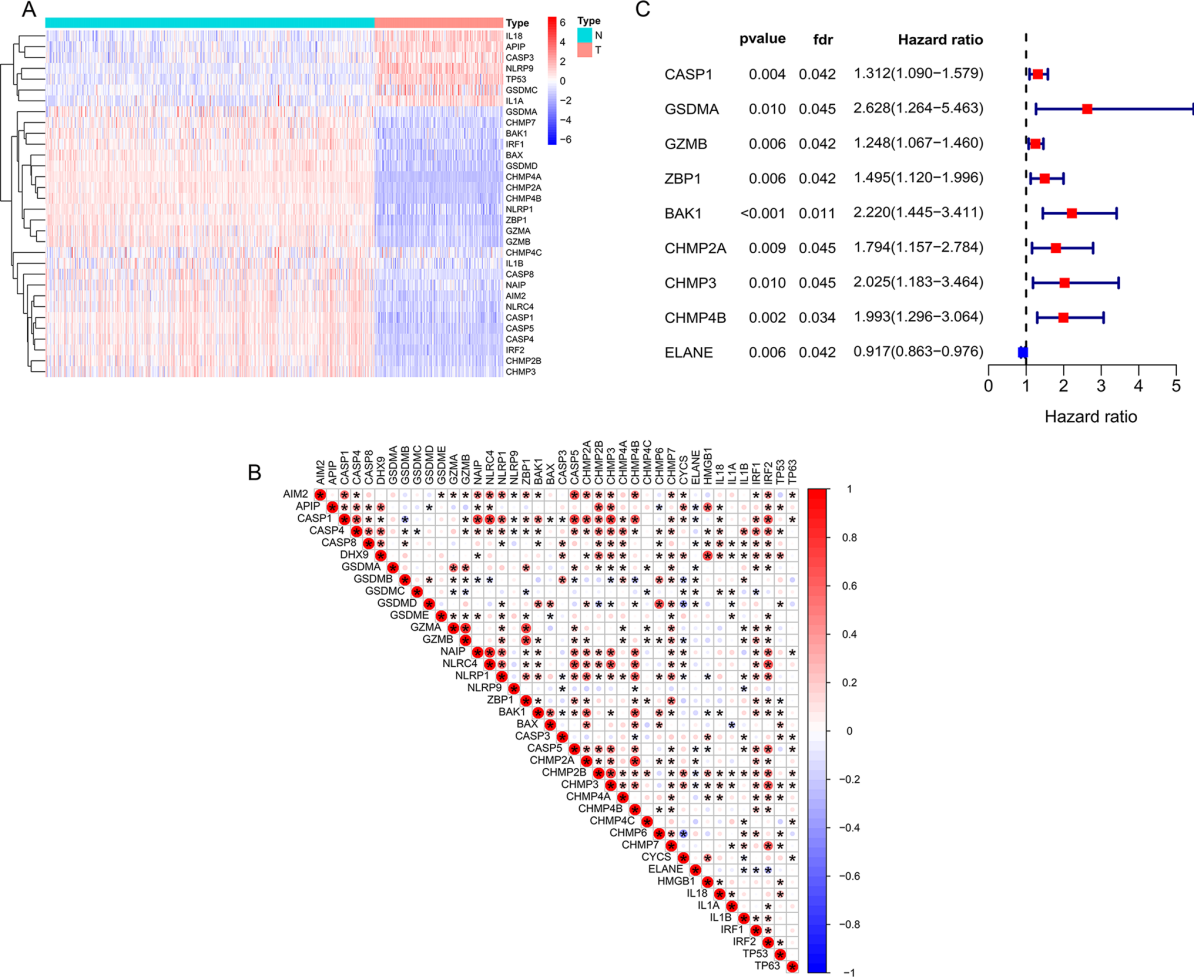
Biological characteristics of pyroptosis-related genes in AML. **A** Landscape of expression level of pyroptosis-related genes in normal samples (n = 386) and AML samples (n = 151). N, normal; T, tumor. **B** The correlation between pyroptosis-related genes in AML samples. *p < 0.05. The darker the color, the stronger the correlation. **C** Univariate analyses were utilized for estimating the impact of pyroptosis-related genes on survival of AML patients. Red, risky factors. Blue, protective factors

The study flowchart was presented in Fig. 1. To screen out pyroptosis genes related to AML tumorigenesis, we compared the expression level of 40 pyroptosis-related genes between AML patient cohort TCGA (n = 151) and healthy people cohort GTEx (n = 386). Heatmap showed that 32 pyroptosis-related genes were abnormally expressed in AML than the healthy, including higher expression of IL18, APIP, CASP3, NLRP9, TP53, GSDMC, IL1A and lower expression of GSDMA, CHMP7, BAK1, IRF1, BAX, GSDMD, CHMP4A, CHMP2A, CHMP4B, NLRP1, ZBP1, GZMA, GZMB, CHMP4C, IL1B, CASP8, NAIP, AIM2, NLRC4, CASP1, CASP5, CASP4, IRF2, CHMP2B and CHMP3 (Fig. 2A). We used Pearson test to evaluate correlations between 40 pyroptosis-related genes in AML from TCGA dataset and found that they were closely related (Fig. 2B). Univariate cox regression analysis showed that 9 pyroptosis-related genes were significantly associated with the prognosis of AML patients. Among them, ELANE [FDR = 0.042, HR (95% CI) 0.917 (0.863–0.976)] was the protective factor as well as CASP1[FDR = 0.042, HR (95% CI) 1.312 (1.090–1.579)], GSDMA [FDR = 0.045, HR (95% CI) 2.628 (1.264–5.463)], GZMB [FDR = 0.042, HR (95% CI) 1.248 (1.067–1.460)], ZBP1 [FDR = 0.042, HR (95% CI) 1.495 (1.120–1.996)], BAK1 [FDR = 0.011, HR (95% CI) 2.220 (1.445–3.411)], CHMP2A [FDR = 0.045, HR (95% CI) 1.794 (1.157–2.784)], CHMP3 [FDR = 0.045, HR (95% CI) 2.025 (1.183–3.464)] and CHMP4B [FDR = 0.034, HR (95% CI) 1.993 (1.296–3.064)] were risk factors for AML (Fig. 2C), Among these genes, only ELANE was identified as a protective prognostic factor. In this group of 9 genes, we aimed to determine whether they remained prognostic factors after adjusting for the effects of age and gender. Therefore, we performed multivariable Cox regression analysis for each gene with age and gender as covariates. The results indicated that CHMP4B, BAK1, CHMP2A, and ELANE remained prognostic factors after adjustment (Figure S1). All the above analysis suggested that the expression of pyroptosis-related genes was different in the AML patients and healthy people, indicating that pyroptosis-related genes play an important role in the occurrence, development and prognosis of AML.

### Identification of different pyroptosis patterns in AML

**Fig. 3:**
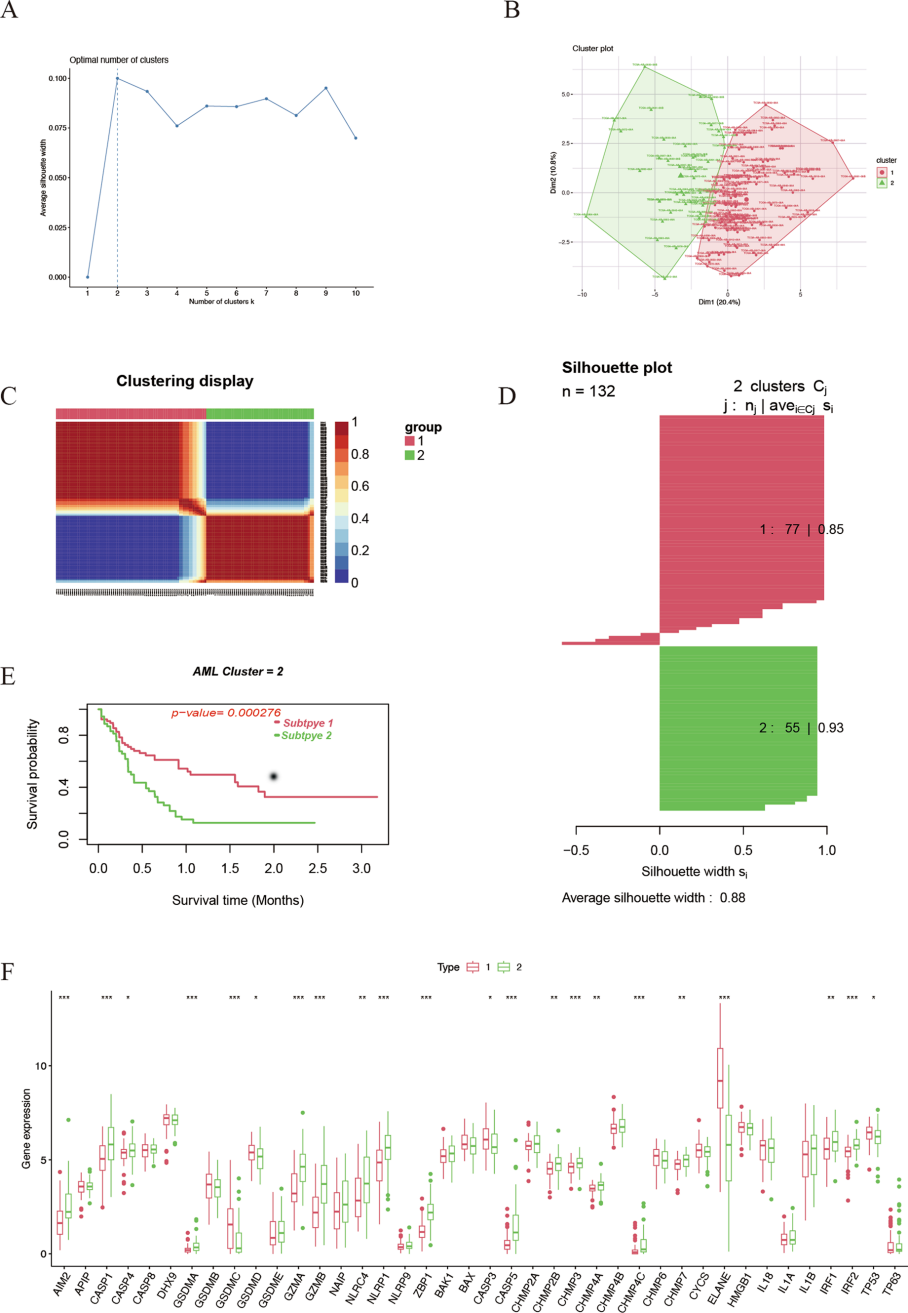
Identification of different AML subtypes. **A** FactoExtra software package was used to determine the optimal number of clusters. **B** Cluster results were visualized with factoextra. **C** Non-negative matrix factorization (NMF) was used for clustering analysis of AML samples. **D** Draw silhouette width plots using CancerSubtypes package. **E** Survival analysis of different AML subtypes was performed using CancerSubtypes package. **F** Box plots were plotted for expression of pyroptosis-related genes in two different AML subtypes. C2 is designated as the ELANE^low^ group, and C1 as the ELANE^high^ group

**Fig. 4:**
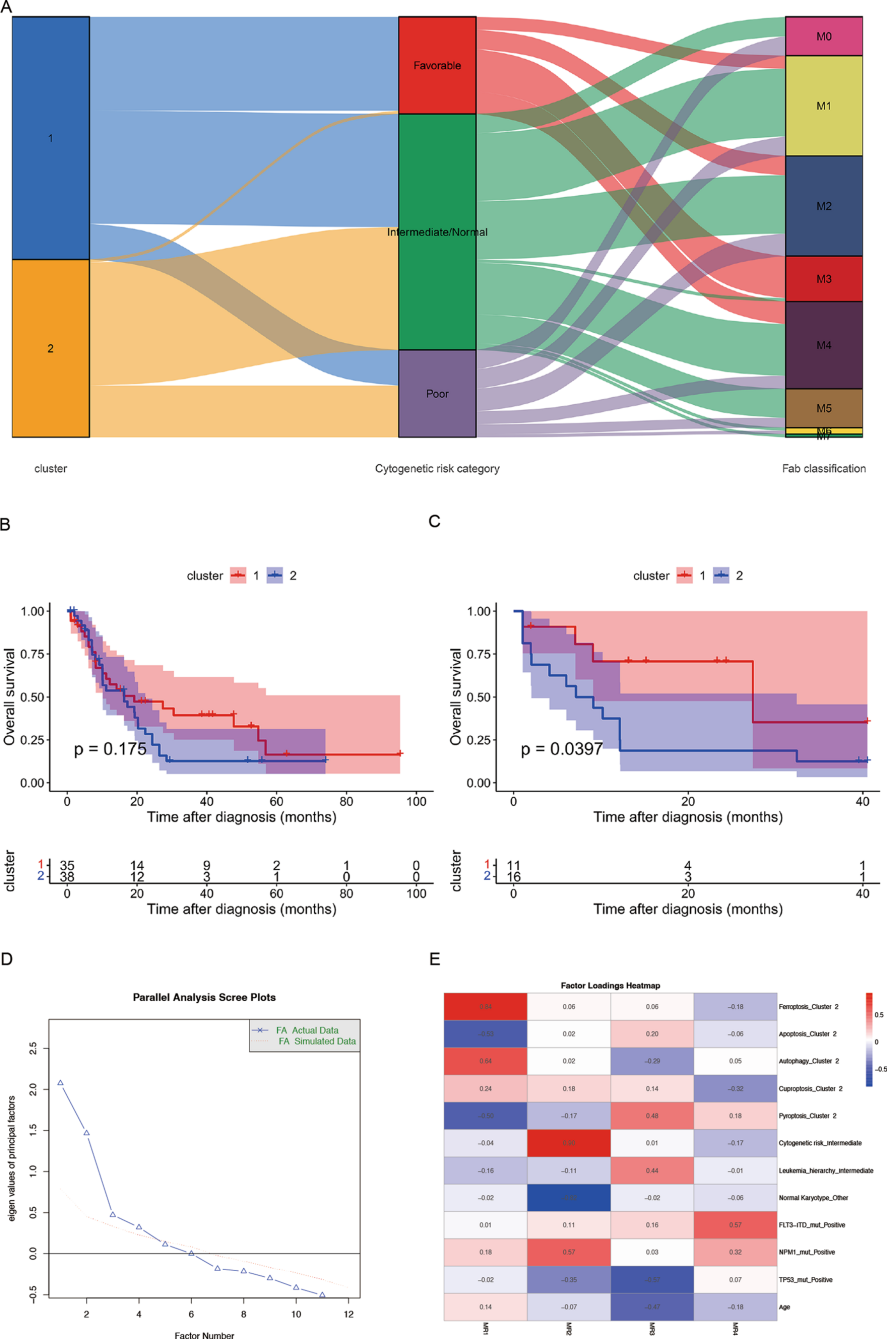
Factor analysis among pyroptosis patterns and other features. **A** Alluvial diagram showing the changes of pyroptosis clusters, cytogenetic risk categories and Fab classification. **B** Survival analysis of two pyroptosis patterns for AML patients with normal karyotype using Kaplan–Meier curves. **C** Survival analyses of two pyroptosis patterns for AML patients with poor karyotype using Kaplan–Meier curves. **D** The scree plot suggests the optimal number of factors to select. **E** The heatmap shows the factor loadings of different features across the four factors. C2 is designated as the ELANE^low^ group, and C1 as the ELANE^high^ group

We used Nonnegative matrix factorization (NMF), an unsupervised clustering method, to stratify AML patients based on the expression of 40 pyroptosis-related genes in TCGA. Factoextra package were utilized to generate the optimal number of clusters and it was 2 (Fig. 3A and B). Two pyroptosis patterns were identified using NMF method (Fig. 3C), with 77 samples in subtype 1 and 55 samples in subtype 2. The silhouette width value was 0.88, indicating a remarkable match between the AML samples and its identified subtypes (Fig. 3D). Notably, the overall survival (OS) rate of AML patients between subtype 1 and subtype 2 were significantly different, and the former exhibited a favorable survival (Fig. 3E). Next, we compared the expression level of pyroptosis-related genes between the two subtypes to verify the rationality of this grouping pattern. As a result, 19 pyroptosis-related genes were differentially expressed in the two subtypes, with most of which were up-regulated in subtype 2 (AIM2, CASP1, GSDMA, GZMA, GZMB, NLRC4, NLRP1, ZBP1, CASP5, CHMP2B, CHMP3, CHMP4A, CHMP4C, CHMP7, IRF1, IRF2) and few were up-regulated in subtype 1 (GSDMC, ELANE, TP53) (Fig. 3F). Subsequently, to validate the grouping, we selected another AML cohort GSE10358 with transcriptome data of microarray to verify the feasibility of dividing AML into two subtypes according to the characteristics of pyroptosis. Notably, consistent with the cluster in TCGA, NMF method showed that the validation group (n = 91) was also stratified into two subtypes, and subtype 1 with a remarkable better prognosis (Figure S2A to S2E). In addition, the transcriptional change trend of CASP1, ZBP1, and ELANE between the two subtypes in the validation set was similar to that of the sample from TCGA, and only ELANE were upregulated in subtype 1 (Figure S2F). Although there was no significant difference, the expression level of AIM2, GZMA, GZMB, NLRC4, NLRP1 and CASP5 was elevated in subtype 2 of validation set, which was consistent with the sample from TCGA. Among these pyroptosis-related genes, the expression difference of ELANE was the most prominent between the two subtypes. Therefore, we named group 1 as the ELANE^high^ pyroptosis group and group 2 as the ELANE^low^ pyroptosis group. The alluvial diagram showed that patients in pyroptosis cluster 2 were primarily characterized by poor or normal karyotypes, whereas those in pyroptosis cluster 1 were predominantly associated with favorable or normal karyotypes (Fig. 4A). Meanwhile, we analyzed the distribution differences of various clinical subgroups between the two groups and determined their significance using Fisher's exact test. There are significant differences in cytogenetic risk (p < 0.001) and age (p = 0.015) between the two subtypes, with ELANE^high^ showing a more favorable cytogenetic risk profile and a higher proportion of younger patients (Supplementary Table 3). However, there is no significant difference in the distribution of sex between the two subtypes (p = 0.27). Further analyses demonstrated that in AML patients with the normal karyotype, there was no significant difference in the overall survival between the two pyroptosis patterns (Fig. 4B); while in the poor karyotypes group, the prognosis of AML patients with high pyroptosis pattern was worse (Fig. 4C).

We aimed to investigate the correlation between pyroptosis expression patterns, other types of cell death, and clinical features. To achieve this, we performed factor analysis on variables including pyroptosis, ferroptosis, apoptosis, autophagy, cuproptosis, karyotype, age, cellular hierarchy composition (stemness), karyotype normality, TP53 mutation, FLT3-ITD, and NPM1 mutations. Similar to pyroptosis, ferroptosis, apoptosis, autophagy, and cuproptosis were subjected to unsupervised clustering analysis in AML samples, resulting in two distinct clusters in the TCGA array. The classification of cellular hierarchy composition was based on results from Andy G. X. Zeng et al. [19]. We utilized the fa.parallel() function to generate a scree plot, which indicated that four factors should be retained (Fig. 4D). In Factor 1, the highest factor loadings were observed for four cell death models: pyroptosis, ferroptosis, autophagy, and apoptosis. Cuproptosis exhibited a relatively even distribution of loadings across multiple factors, with the highest loading in Factor 4, which also included FLT3-ITD. Pyroptosis showed the highest loading in Factor 1 but also a notable loading in Factor 3, which additionally included intermediate stemness, TP53 mutation, and age. The primary loading variables for Factor 2 were intermediate-risk karyotype, normal karyotype, and NPM1 mutation (Fig. 4E). Factor analysis revealed a strong correlation between the pyroptosis classification pattern and the other three cell death models (ferroptosis, autophagy, and apoptosis), with pyroptosis primarily associated with Factor 1. Moreover, the pyroptosis pattern was found to be linked with age, TP53 mutation, and leukemia stemness, as reflected in Factor 3. These findings suggest that the pyroptosis pattern in AML may represent a clinical manifestation influenced by multiple factors, including karyotype, age, leukemia stemness, or interactions with other cell death modes.

### Immunological characteristics of the two distinct pyroptosis patterns

**Fig. 5:**
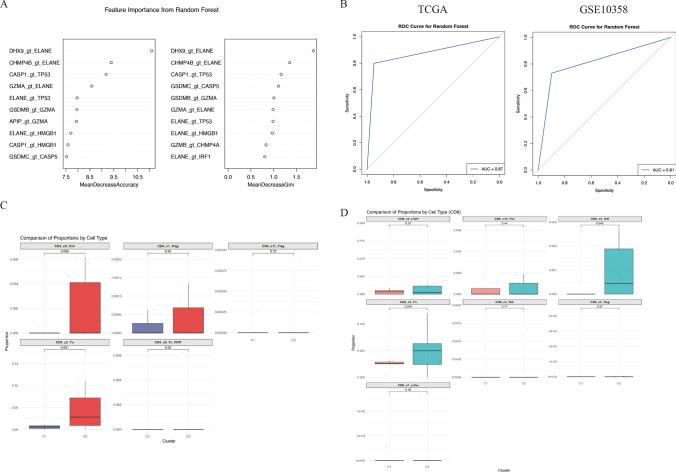
Differences in the immune microenvironment between two distinct pyroptosis subtypes. **A** A gene pair random forest model was trained to classify pyroptosis subtypes, with the top 10 most important variables displayed. The left plot ranks the variables by mean decrease accuracy, while the right plot uses mean decrease Gini. **B** Receiver operating characteristic (ROC) curves were used to assess the accuracy of the random forest model in predicting pyroptosis subtypes, with results shown for TCGA and GSE10358 cohorts on the left and right, respectively. **C** Faceted boxplots illustrate the differences in the percentage of various CD4 + T cell subtypes between the two pyroptosis subtypes. **D** Faceted boxplots depict the differences in the percentage of various CD8 + T cell subtypes between the two pyroptosis subtypes. C2 is designated as the ELANE^low^ group, and C1 as the ELANE^high^ group

We aimed to investigate whether there are differences in the immune microenvironment between two distinct pyroptosis patterns. The dataset GSE116256 contains single-cell RNA sequencing data from 16 newly diagnosed AML patients. One sample was excluded due to insufficient tumor cell numbers, leaving data from 15 patients for subsequent analysis. We sought to classify these 15 patients according to their pyroptosis patterns, but due to batch effects and differences in data types, we were unable to use gene expression alone to train a pyroptosis classification model. Therefore, we developed a gene pair random forest model utilizing the inherent size relationships among 40 pyroptosis-related genes. The relative size of gene expressions was used as input features, such as Gene 1 > Gene 2, where the feature is set to 1 if true and 0 if false. This approach generated 40*39/2 features from the 40 pyroptosis genes, with the grouping data of pyroptosis patterns being the target for fitting. The model was trained using TCGA data. Among the features, the pair DHX9 > ELANE had the highest importance (Fig. 5A). The AUC of the prediction results in the TCGA training set was 0.87, and the model also demonstrated strong predictive accuracy with an AUC of 0.81 in the validation set, GSE10358 (Fig. 5B). Notably, GSE10358, consisting of microarray data, differs significantly from TCGA's next-generation sequencing data in terms of data measurement methods; nonetheless, the model exhibited strong predictive performance in GSE10358 as well.

Subsequently, we aggregated the tumor cell expression data from the 15 single-cell samples to obtain total counts, analogous to the bulk sequencing total count, and normalized it to CPM. We then applied the random forest model to predict the pyroptosis pattern in these 15 samples. Five samples were classified into group ELANE^high^, while the remaining 10 samples were assigned to group ELANE^low^ (Supplementary Table 4). Using the original cell annotations, we analyzed the differences in the proportions of various cell types between the two groups (Figure S3A). Notably, B cells and cytotoxic T lymphocytes (CTLs) were significantly upregulated in the ELANE^low^ group. As the authors of the original study did not further subtype the T cells, we utilized a T cell atlas integrated by Yanshuo Chu et al. [20] to further classify the T cells. We employed the MapQuery function from Seurat to map the AML T cell data onto the pre-established T cell atlas (divided into CD4 + and CD8 + T cells) in UMAP space, and predicted the cell types based on the corresponding reference data (Figure S3B-C). In the CD4 + T cell subset, the proportion of naive T cells was significantly increased in ELANE^low^, and the proportion of central memory T cells was elevated in ELANE^low^ (p = 0.056) (Fig. 5C). In the CD8 + T cell subset, the proportions of naive T cells and effector T cells were significantly higher in ELANE^low^ (Fig. 5D). Overall, group ELANE^low^ exhibited a stronger adaptive immune response, suggesting that group ELANE^low^ may possess a higher immunogenic potential.

At the same time, we analyzed the expression of CASP1, ELANE, and ZBP1 in the microenvironment of AML patients. Single-cell transcriptome data revealed that CASP1, ELANE, and ZBP1 were predominantly expressed in monocytes, GMP cells, and plasma cells, respectively (Figure S3D). Furthermore, compared to normal samples, the proportion of monocytes expressing CASP1 was significantly increased, and the average expression level of CASP1 was higher in AML patients. In contrast, the proportion of GMP cells expressing ELANE and plasma cells expressing ZBP1 was significantly decreased, with both showing lower average expression levels (Figure S4).

**Fig. 6:**
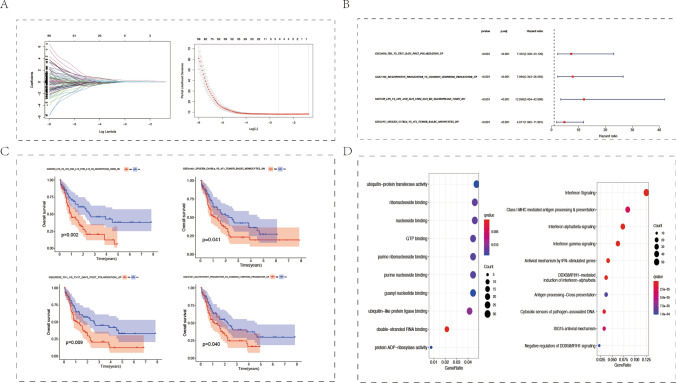
Prognostic gene sets in AML samples. **A** Least absolute shrinkage and selection operator (LASSO) coefficient profiles (y-axis) of the gene sets and the optimal penalization coefficient (λ) via threefold cross-validation based on partial likelihood deviance. The dotted vertical lines represented the optimal values of λ. The top x-axis indicated the numbers of gene sets, and the lower x-axis showed the log (λ). **B** Univariate analyses were utilized for estimating the impact of four immunologic gene sets on prognosis of AML patients. **C** Enrichment degree of gene sets was negatively correlated with overall survival time. **D** Analysis of Molecular Function (MF) and Reactome pathway. Four prognostic immune gene sets were analyzed by MF enrichment (left panel) and Reactome pathway (Right Panel)

Subsequently, we compared the immune characteristics of two AML patterns with different levels of pyroptosis and identified immune-related prognostic genes. We compared enrichment score (ES) of 5219 immune gene sets between the two pyroptosis patterns to further explore the underlying immunity mechanism. Figure S5A showed that 998 immune gene sets were highly enriched in ELANE^low^ group and only 431 immune gene sets were highly enriched in subtype ELANE^high^ in TCGA. As for GSE10358, most immune gene sets were with a higher ES in subtype ELANE^low^ (Figure S5A), indicating that pyroptosis may be positively associated with immunity. We acquired 311 overlap gene gets enriched in the high pyroptosis pattern subtype ELANE^low^ via intersecting enriching gene sets of GSE10358 and TCGA (Figure S5B), which exhibited high overlap. Next, we extracted four prognosis associated gene sets from the 311 gene sets highly enriched in subtype ELANE^low^ by lasso method (Fig. 6A), including GSE26030_TH1_vs_TH17_DAY5_POST_POLARIZATION_UP, GSE37301_MULTIPOTENT_PROGENITOR_vs_COMMON_LYMPOID_PROGENITOR_UP, GSE5589_LPS_vs_LPS_AND_IL10_STIM_IL10_KO_MACROPHAGE_45MIN_DN and GSE21297_SPLEEN_C57BL6_vs_4T1_TUMOR_BALBC_MONOCYTES_DN which were associated with helper T cell (Th), IL10, progenitor and myeloid-derived suppressor cells (MDSCs). Univariate cox regression analysis showed that all the four gene set were risk factors for AML (Fig. 6B). In addition, The K-M OS survival curve showed that the enrichment degree of each gene set was negatively correlated with overall survival time (Fig. 6C). We analyzed all the genes from the four prognostic immune gene sets by MF enrichment and Reactome pathway to further explore the potential mechanisms of immune effect on the prognosis of AML. All the genes were mainly associated with terms of cell adhesion molecule binding, ubiquitin protein ligase binding, cytokine activity in MF, and interferon signaling, class I MHC mediated antigen processing & presentation in Reactome pathway, respectively (Fig. 6D).

### Protein–protein interaction network and hub genes

**Fig. 7:**
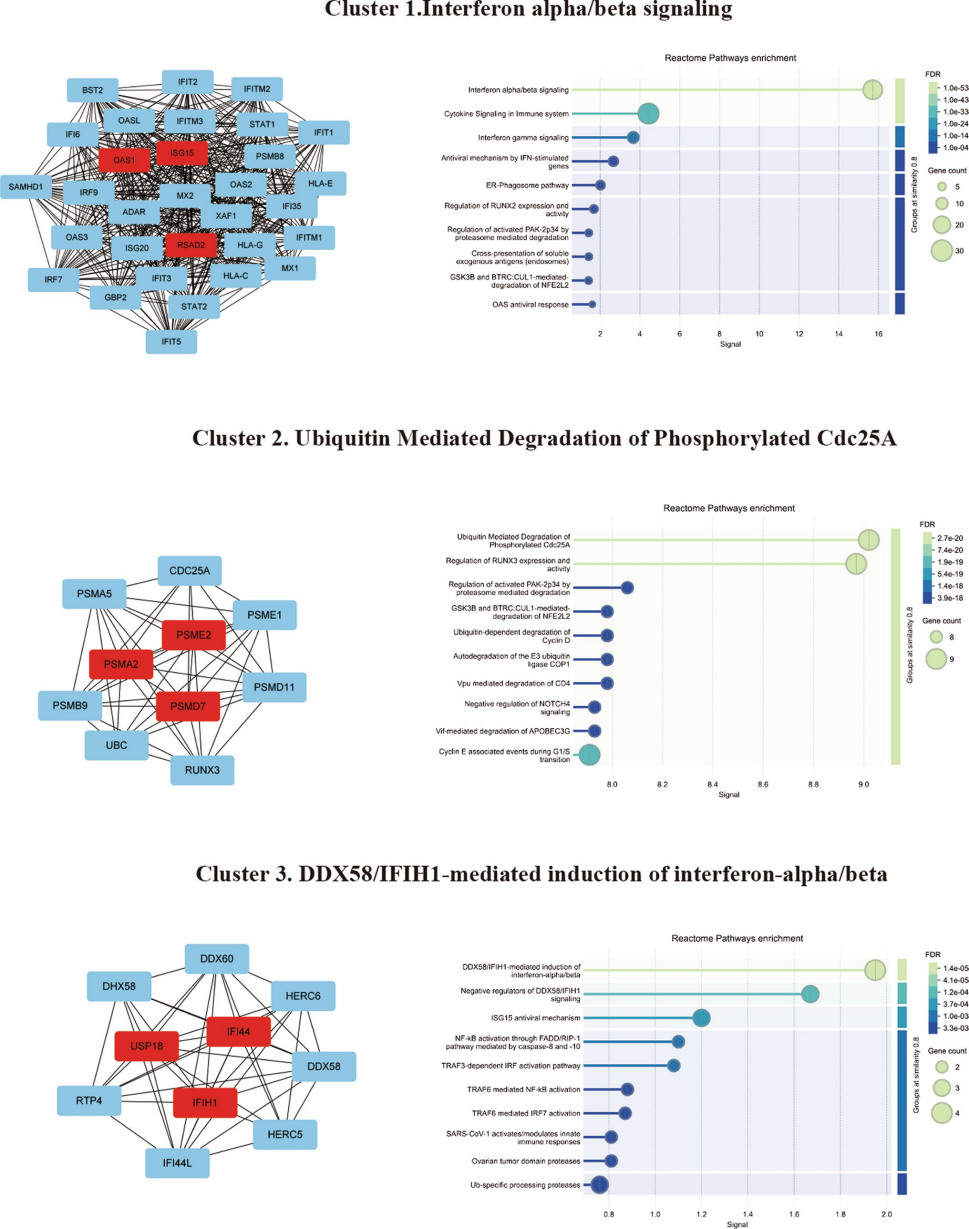
The protein–protein interaction (PPI) network of four prognostic immune gene sets. The top three clusters and hub genes (red) were obtained by the Molecular Complex Detection (MCODE) plug-in of Cytoscape. The right panel shows the Reactome pathway enrichment results for each gene cluster, with the pathway exhibiting the strongest signal used for cluster naming

To identify hub genes from the four prognostic gene sets, we extracted all the genes from the four prognostic gene sets and constructed a protein–protein interaction network. The top three clusters were obtained by the Molecular Complex Detection (MCODE) plug-in of Cytoscape. We performed Reactome pathway analysis to annotate the three clusters and named each cluster based on the pathway with the strongest signal strength. Cluster 1 was named "Interferon alpha/beta signaling," Cluster 2 was named "Ubiquitin Mediated Degradation of Phosphorylated Cdc25A," and Cluster 3 was named "DDX58/IFIH1-mediated induction of interferon-alpha/beta." In addition, we calculated the hub genes in each cluster by values of degree. The top three hub genes in cluster 1 were OAS1, ISG15 and RSAD2, cluster 2 were PSMA2, PSME2 and PSMD7, cluster 3 were USP18, IFI44 and IFIH1 (Fig. 7). Finally, after adjusting for age and gender, Cox regression analysis identified three hub genes (PSME2, ISG15, OAS1) from the nine key genes in the four prognostic gene sets as potential prognostic biomarkers for AML (Figure S6).

## Discussion

Pyroptosis, a new form of programmed cell death, was associated with inflammation, immunity, and the development of tumor through activation of inflammasome, including multiple myeloma [21], lung cancer [22], gastric cancer [23], cervical cancer [24] and colorectal cancer [25]. Ding et al. demonstrated that pyroptosis inducers exhibited antitumor immunity activity by inhibiting tumor growth and metastasis [26]. However, the fundamental role of pyroptosis in the pathogenesis, development and treatment of AML remains to be revealed. Here, we revealed that the transcriptional level of pyroptosis-related genes was altered in AML by bioinformatic analysis. In addition, AML patients were clustered into two subtypes according to the expression level of 40 pyroptosis-related genes. In addition, applying the same unsupervised clustering method to the GSE10358 array, we identified two distinct clusters. A comparison of gene expression patterns between the two groups revealed that the C1 and C2 clusters in GSE10358 were highly similar to those in the TCGA dataset. However, a notable difference was that fewer significantly differentially expressed genes were observed between the two groups in GSE10358. Despite this, the expression trends of several key genes were consistent across both datasets. For instance, ELANE was significantly downregulated in Group 2, while AIM2, CASP1, and ZBP1 were significantly upregulated in the same group. These findings suggest that these genes, particularly ELANE, could serve as marker genes for characterizing the pyroptosis pattern in AML. Therefore, we designated C1 as the ELANE^high^ group and C2 as the ELANE^low^ group. Furthermore, the enrichment scores (ES) of immune-related gene sets between the two AML subtypes showed significant differences, with four of these sets being linked to AML prognosis.

Pyroptosis-related genes were abnormally expressed in AML and were related to the survival outcome of patients. In our analysis, pyroptosis-related factor IL18 were elevated in the peripheral blood samples of 132 AML patients from TCGA database, which were consistent with the research of Jia et al. [6]. We found that CASP3 was significantly upregulated in AML. In addition, Yang et al. established the heteroplastic AML mouse model using THP-1 cells and demonstrated that vitamin B6 triggered the lysis of GSDME by activating CASP3, induced noncanonical pyroptosis of leukemic THP-1 cells in mice, and could also induce pyroptosis of primary AML cells in vitro, exerting anti-AML activity. Similarly, another study confirmed that ardisianone induced pyroptosis of HL60 cells through activation of CASP3 and CASP1 and promoted the differentiation of leukemic cells into monocytes and macrophages [27]. All of the above confirmed the protective role of CASP3 in AML. In contrast to our findings that CASP1 was a risky factor for AML patients, Johnson et al. demonstrated that DPP8/9 inhibitor induced death of AML cells through CASP1-dependent pyroptosis [28]. GSDMD, an important protein mediating the lysis of cytomembrane in pyroptosis, were found to be down-regulated in AML in our bioinformatic study. However, Ahn et al. showed that arsenic inhibited the formation of Asc pyroptosome and cleavage of GSDMD, and reduced expression of PML-RARA in acute promyelocytic leukemia (APL), indicating that GSDMD may promoted the development of APL [29]. GZMB was found to be a risk factor for AML and its hypermethylation was associated with inferior overall survival [30]. The expression level of GZMA was positively correlated with GZMB, but its role in AML has not been reported. In conclusion, pyroptosis was closely associated with the progress of AML. However, the specific role of pyroptosis in the occurrence, development and treatment of AML needs to be further investigated.

The concise classification method is conducive to the target treatment of AML. According to the genome-wide methylation profiles of cancer samples, Gao et al. developed three AML subtypes with different outcomes and identified their marker genes [31]. In addition, metabolism-related genes, ferroptosis-related genes, immune-related genes and DNA repair genes could become potential prognostic biomarkers for AML [32–34]. Here, based on the activity changes of pyroptosis-related genes in AML samples, we stratified AML into two subtypes. These two groups exhibited differential survival rates, indicating that the baseline pyroptosis levels in cancer cells from newly diagnosed AML patients are significantly associated with prognosis. Current research on pyroptosis in AML primarily focuses on its role in drug-induced cancer cell death. Our study, however, reveals the presence of two distinct baseline patterns in untreated samples. Factor analysis further suggests that the pyroptosis pattern in AML may be interconnected with other cell death pathways, such as ferroptosis, apoptosis, and autophagy. Notably, compared to other death pathways, the pyroptosis pattern shows the strongest association with AML cytogenetics and stemness. This suggests that variations in the pyroptosis pattern may be linked to genomic features and leukemia stem cell characteristics.

Pyroptosis is closely related to immunity, and both play important roles in the genesis and development of AML. Through single-cell analysis of different pyroptosis patterns, we found that the ELANE^low^ pyroptosis group exhibited a significantly higher proportion of T cells and B cells. Additionally, single-cell transcriptomic data revealed that three pyroptosis-related genes, CASP1, ELANE, and ZBP1, were differentially expressed in the immune microenvironment of healthy individuals and AML patients. CASP1, a key molecule in the classical pyroptosis pathway, was predominantly expressed in monocytes. Guo et al. reported that inflammatory monocytes could be activated by abnormal IL-36 production in AML, which in turn blocked CD8 T cell-mediated leukemia clearance and promoted leukemic progenitor cell growth [35]. Therefore, we inferred that CASP1 may be involved in the development of AML by influencing the function of monocytes. Another important pyroptosis-related gene ELANE was mostly expressed in granulocyte macrophage progenitors (GMPs). Disruption of GMP formation by deleting the lineage-restricted transcription factor C/EBPa inhibited normal granulocyte formation and prevented initiation of AML [36]. Second, we found that 311 immune gene sets were commonly enriched in ELANE^low^ subtype, indicating the close relationship between pyroptosis and immunity in AML, which have ever been reported in colon cancer and cervical cancer [37, 38]. 4 of 311 immunity-related gene sets associated with IL10, helper T cell (Th), progenitor and MDSCs were found to be related with poor prognosis in AML patients. High level IL10 released by regulatory T cells could elevated the stemness of AML cells by activating the PI3K/AKT signal pathway and helped AML cells evade immune surveillance [39]. Th17 cells secreted signature cytokine IL-17A and induced proliferation of IL-17 receptor (IL-17R) positive AML cells. AML patients with a high proportion of Th17 cells had a poor prognosis, while patients with a high proportion of Th1 cells had longer survival [40]. MDSCs activated by pyroptosis-related genes might creat an immunosuppressive microenvironment and promote AML development [41, 42]. Third, we conducted enrichment analysis of all the genes from the 4 immunity-related prognostic gene sets and found that they were related to ribonucleoside binding, nucleoside binding, purine necleoside binding and so on, which might worsen the prognosis by disturbing DNA synthesis and damage repair in AML. Finally, nine hub genes were identified from the four immunity-related prognostic gene sets, two of which, including OAS1 and ISG15, have been shown to be associated with the prognosis of AML, as reported in previous studies [43, 44], consistent with our findings. In addition, ISG15 and IFIH1, as the stimulators of interferon signaling, may promote the development of AML by activating pyroptosis-related genes [42, 45–47].

However, there are some limitations in this study. Firstly, the results of our analysis should be validated from more online datasets. Secondly, the identified pyroptosis-related genes should be confirmed in vitro experiments or through clinical samples. Therefore, in future studies, we will continue to investigate the role of these key genes in AML in a more systematic and in-depth manner based on the results of the bioinformatics study as well as the results of the validation in clinical samples.

## Conclusions

In conclusion, we identified two AML subtypes based on the expression levels of pyroptosis-related genes. We further explored the prognostic differences, immune microenvironment variations, and potential associations with other cell death pathways, genomic alterations, and leukemia stemness between these subtypes. Finally, through GSVA pathway analysis and PPI network identification, we uncovered key hub genes. These findings provide new strategies for personalized treatment in AML.

## Supplementary Information

Supplementary Material 1: Figure S1. Pyroptosis genes significantly associated with prognosis after adjusting for age and gender. Nine forest plots depict the results of multivariable Cox regression analysis for nine pyroptosis genes.

Supplementary Material 2: Figure S2. Another validation group (n=91) was selected to verify the feasibility of dividing AML into two subtypes. (A) FactoExtra software package was used to determine the optimal number of clusters. (B) Cluster results were visualized with factoextra. (C) Non-negative matrix factorization (NMF) was used for clustering analysis of AML samples. (D) Draw silhouette width plots using CancerSubtypes package. (E) Survival analysis of different AML subtypes was performed using CancerSubtypes package. (F) Box plots were plotted for expression of pyroptosis-related genes in two different AML subtypes of the validation group. C2 is designated as the ELANE^low^ group, and C1 as the ELANE^high^ group.

Supplementary Material 3: Figure S3. Differences in the immune microenvironment between two pyroptosis subtypes. (A) Faceted boxplots depict the differences in the distribution of 21 immune cell types between the two pyroptosis subtypes. (B) CD4+ T cells in AML patients were mapped to the reference CD4+ T cell atlas. The left panel shows the UMAP plot of the original atlas, while the right panel shows the UMAP positions of AML CD4+ T cells mapped to the reference atlas. (C) CD4+ T cells in AML patients were also mapped to the reference CD8+ T cell atlas. The left panel shows the UMAP plot of the original atlas, while the right panel shows the UMAP positions of AML CD8+ T cells mapped to the reference atlas. (D) Scatter show the expression proportions and average expression levels of three genes in different kinds of immune cells, which is a pooled analysis for cells of healthy donors and AML patients. C2 is designated as the ELANE^low^ group, and C1 as the ELANE^high^ group.

Supplementary Material 4: Figure S4.The expression differences of pyroptosis-related genes across different cell types. (A) Cluster analysis of cell populations in human hematopoietic system. (B) Expression of CASP1 in monocytes. (Left) Source of immune cells of the healthy (blue) and AML patients (red); (Middle) Expression level of CASP1 in monocytes; (Right) Scatter show the expression proportions and average expression levels of CASP1 in monocytes. The color of the dots represents the average expression level of the CASP1, and the area of the dots represents the percentage of monocytes expressing the gene in AML patients. (C) Expression level of ELANE in GMP cells. (D) Expression level of ZBP1 in plasma cells.

Supplementary Material 5: Figure S5. GSVA analysis of immunologic gene sets in AML. (A) Heatmap of enrichment scores for immunologic gene sets in two AML subtypes from TCGA and GSE10358. Each row represents a gene sets, and each column represents a sample. (B) The differential enrichment score of gene sets between the two subtypes from TCGA and GSE10358 were intersected. C2 is designated as the ELANE^low^ group, and C1 as the ELANE^high^ group.

Supplementary Material 6: Figure S6. Core genes significantly associated with prognosis after adjusting for age and gender. Nine core genes were identified through PPI network analysis, with one gene unavailable in TCGA. Multivariable Cox regression analysis was performed using the remaining eight genes. The forest plot in the figure presents the results.

Supplementary Material 7.

## Data Availability

The datasets analyzed during this study are available in the Genotype-Tissue Expression (GTEx) database, the Cancer Genome Atlas (TCGA) database, Gene Expression Omnibus (GEO) database and Gene Set Enrichment Analysis (GSEA) website (https://www.gsea-msigdb.org/gsea/msigdb/index.jsp).
