# Supplementary figures and images for: Identification and validation of pyroptosis patterns in AML via comprehensive bioinformatics analysis
Source: Discov Oncol. 2025 Apr 10;16:509. doi: 10.1007/s12672-025-02298-5 (PMC11985831; doi:10.1007/s12672-025-02298-5)

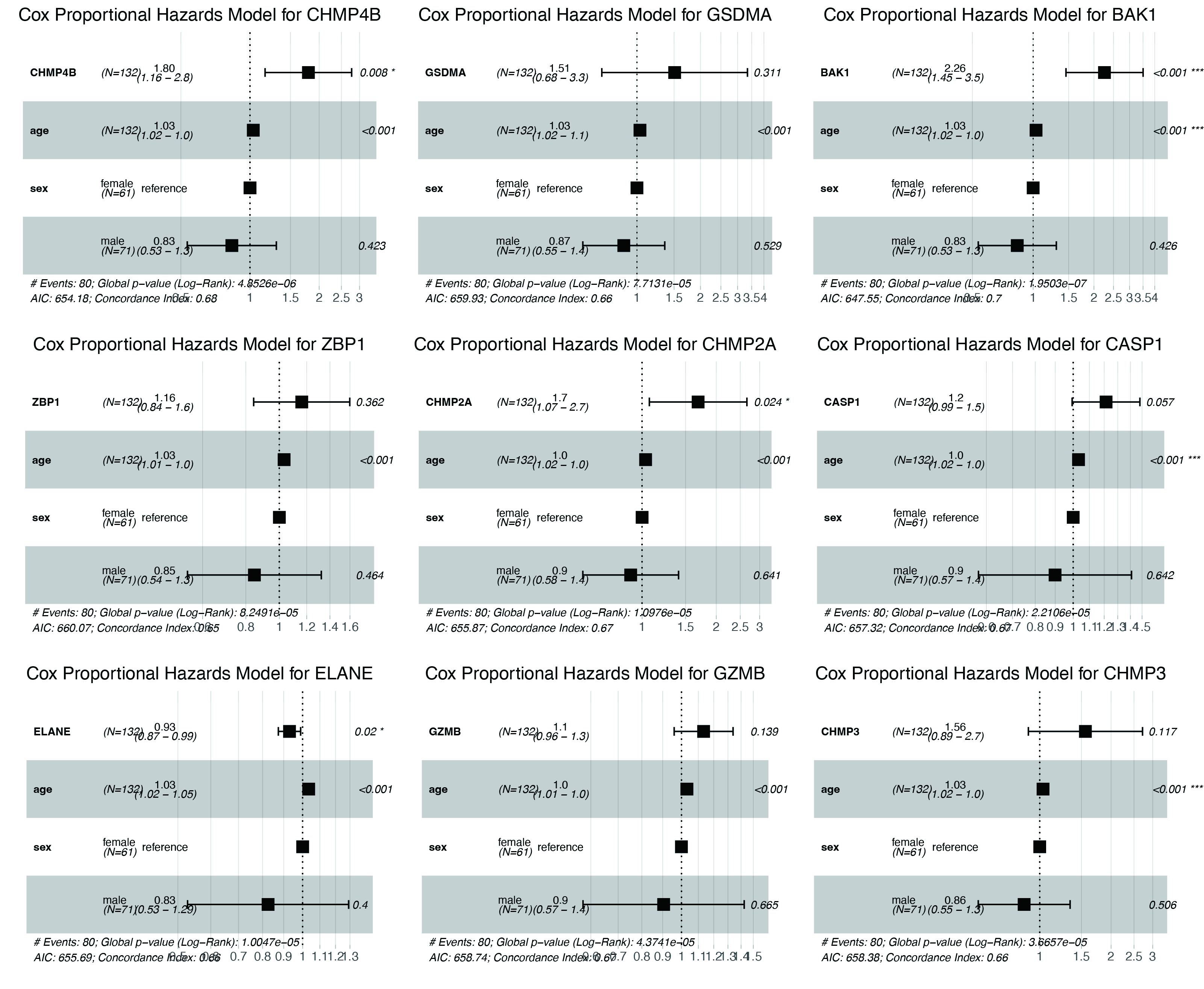

Supplement: Supplementary file 1 — Supplementary Material 1: Figure S1. Pyroptosis genes significantly associated with prognosis after adjusting for age and gender. Nine forest plots depict the results of multivariable Cox regression analysis for nine pyroptosis genes. [file 12672_2025_2298_MOESM1_ESM.tif]

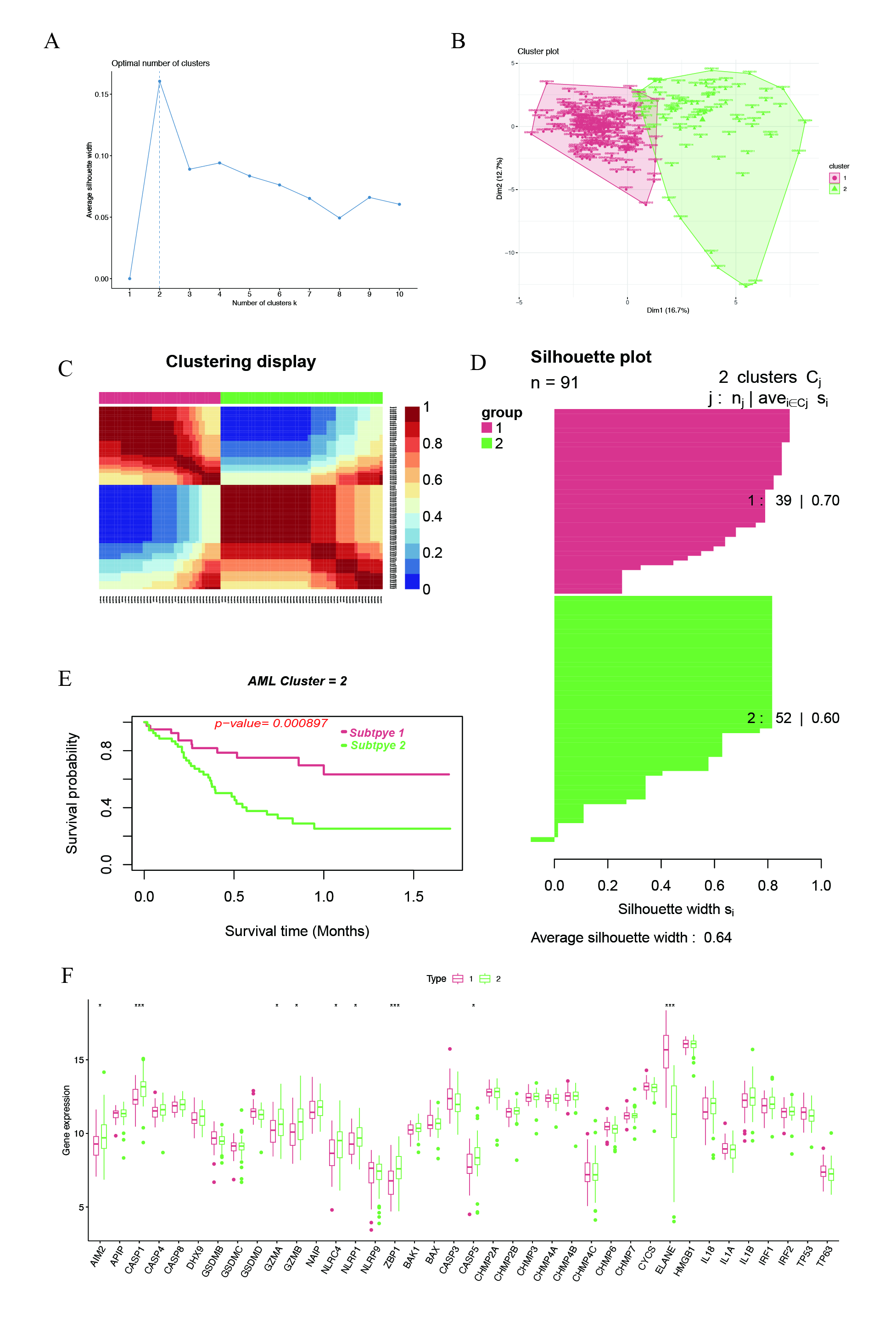

Supplement: Supplementary file 2 — Supplementary Material 2: Figure S2. Another validation group (n=91) was selected to verify the feasibility of dividing AML into two subtypes. (A) FactoExtra software package was used to determine the optimal number of clusters. (B) Cluster results were visualized with factoextra. (C) Non-negative matrix factorization (NMF) was used for clustering analysis of AML samples. (D) Draw silhouette width plots using CancerSubtypes package. (E) Survival analysis of different AML subtypes was performed using CancerSubtypes package. (F) Box plots were plotted for expression of pyroptosis-related genes in two different AML subtypes of the validation group. C2 is designated as the ELANElow group, and C1 as the ELANEhigh group. [file 12672_2025_2298_MOESM2_ESM.tif]

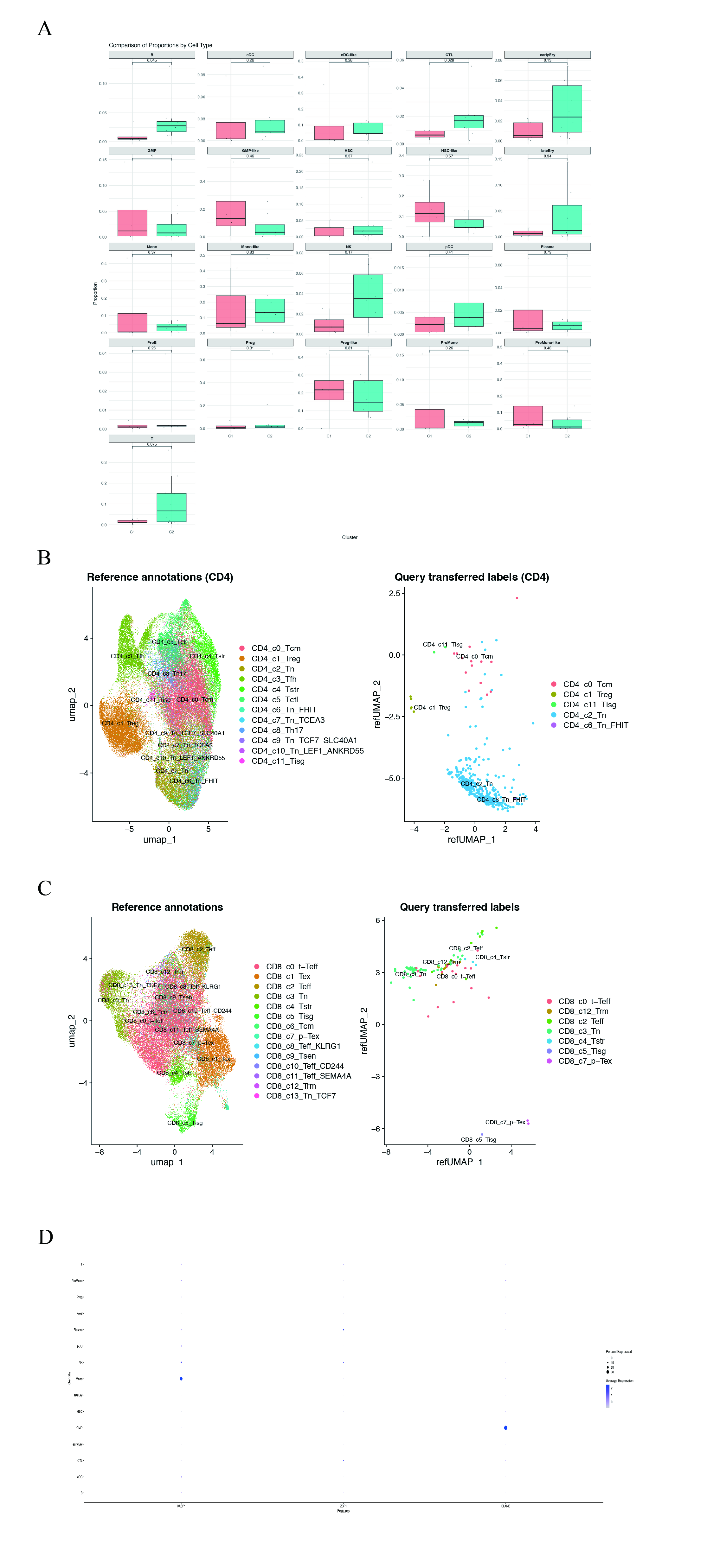

Supplement: Supplementary file 3 — Supplementary Material 3: Figure S3. Differences in the immune microenvironment between two pyroptosis subtypes. (A) Faceted boxplots depict the differences in the distribution of 21 immune cell types between the two pyroptosis subtypes. (B) CD4+ T cells in AML patients were mapped to the reference CD4+ T cell atlas. The left panel shows the UMAP plot of the original atlas, while the right panel shows the UMAP positions of AML CD4+ T cells mapped to the reference atlas. (C) CD4+ T cells in AML patients were also mapped to the reference CD8+ T cell atlas. The left panel shows the UMAP plot of the original atlas, while the right panel shows the UMAP positions of AML CD8+ T cells mapped to the reference atlas. (D) Scatter show the expression proportions and average expression levels of three genes in different kinds of immune cells, which is a pooled analysis for cells of healthy donors and AML patients. C2 is designated as the ELANElow group, and C1 as the ELANEhigh group. [file 12672_2025_2298_MOESM3_ESM.tif]

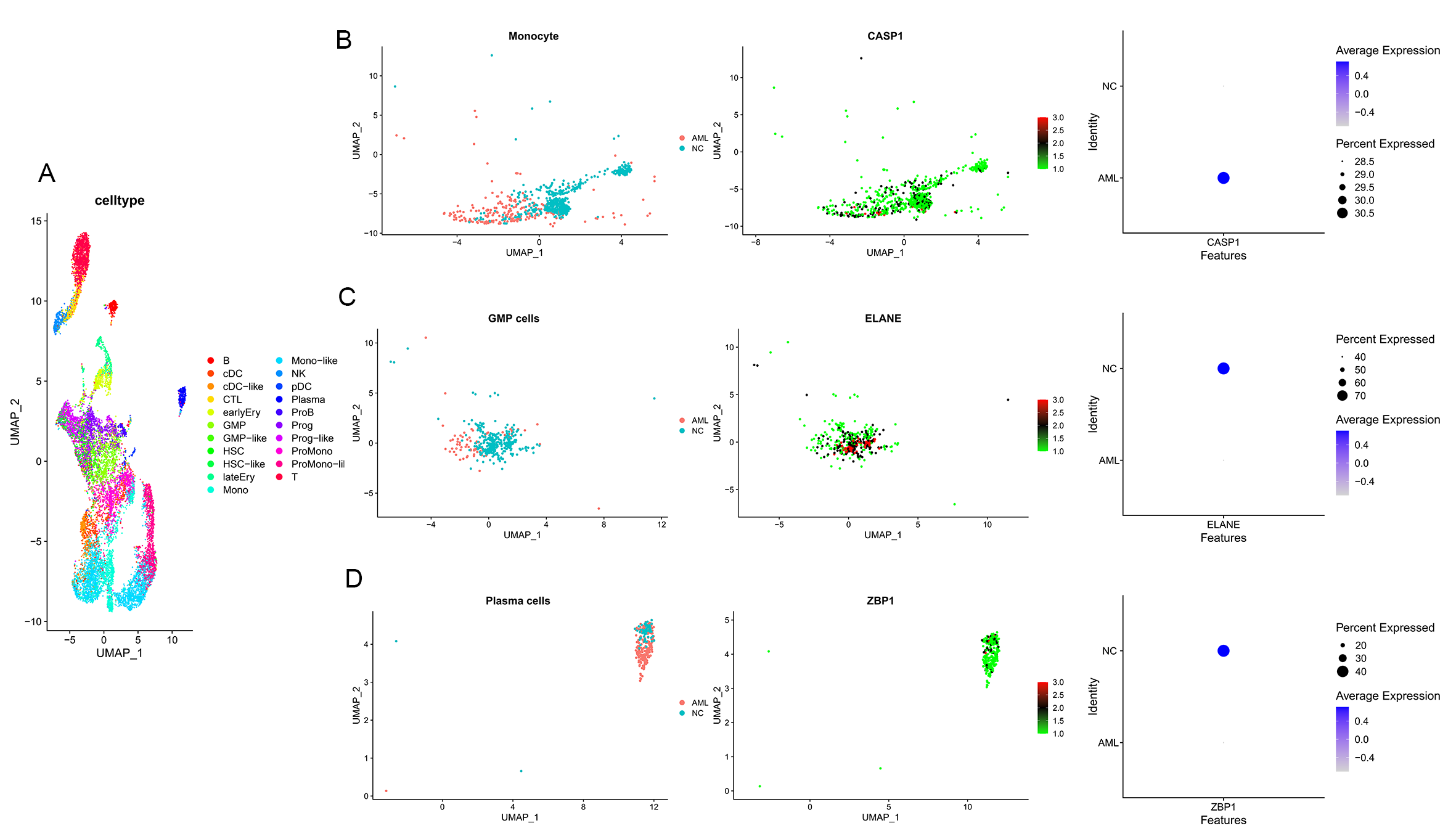

Supplement: Supplementary file 4 — Supplementary Material 4: Figure S4.The expression differences of pyroptosis-related genes across different cell types. (A) Cluster analysis of cell populations in human hematopoietic system. (B) Expression of CASP1 in monocytes. (Left) Source of immune cells of the healthy (blue) and AML patients (red); (Middle) Expression level of CASP1 in monocytes; (Right) Scatter show the expression proportions and average expression levels of CASP1 in monocytes. The color of the dots represents the average expression level of the CASP1, and the area of the dots represents the percentage of monocytes expressing the gene in AML patients. (C) Expression level of ELANE in GMP cells. (D) Expression level of ZBP1 in plasma cells. [file 12672_2025_2298_MOESM4_ESM.tif]

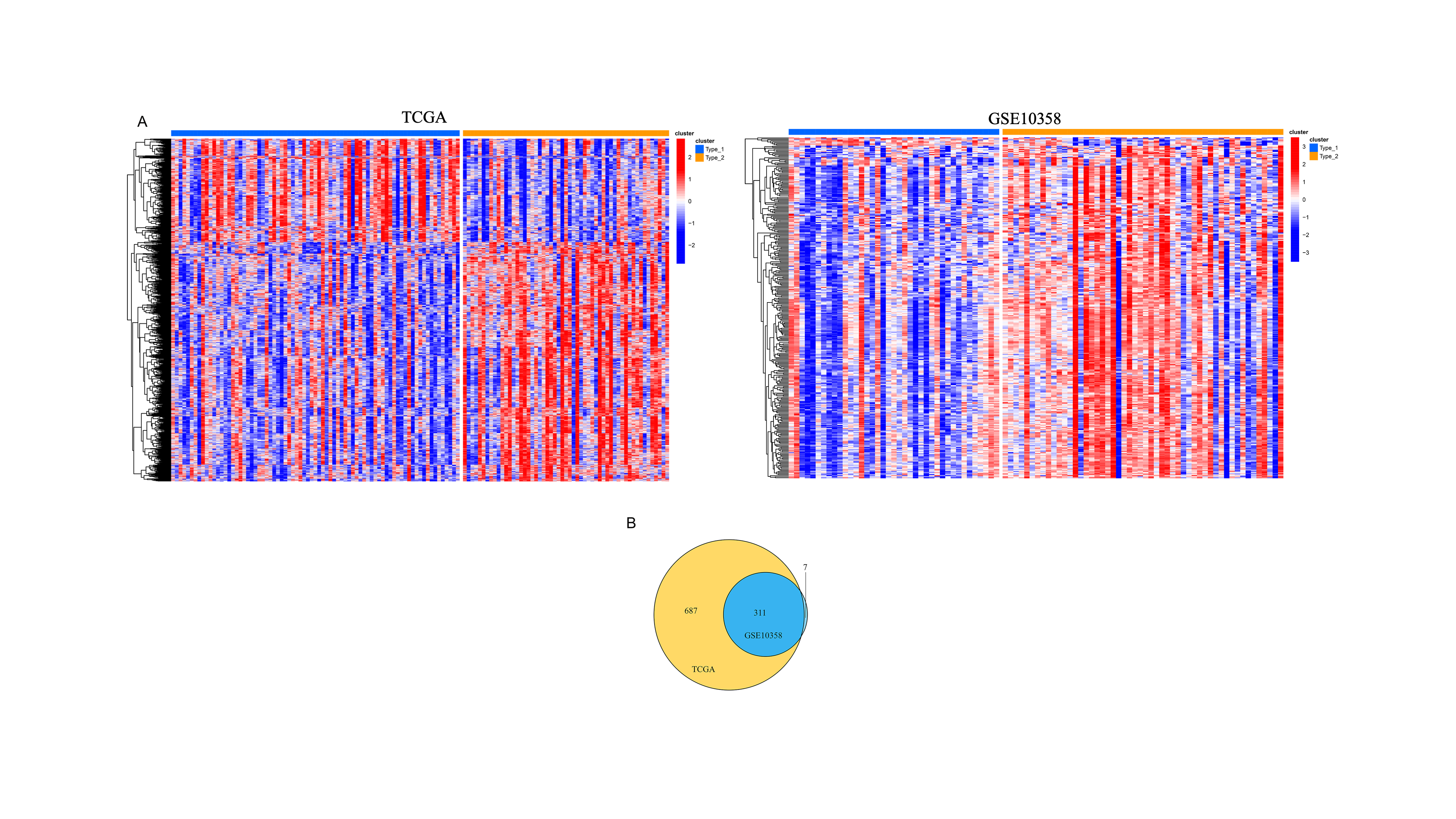

Supplement: Supplementary file 5 — Supplementary Material 5: Figure S5. GSVA analysis of immunologic gene sets in AML. (A) Heatmap of enrichment scores for immunologic gene sets in two AML subtypes from TCGA and GSE10358. Each row represents a gene sets, and each column represents a sample. (B) The differential enrichment score of gene sets between the two subtypes from TCGA and GSE10358 were intersected. C2 is designated as the ELANElow group, and C1 as the ELANEhigh group. [file 12672_2025_2298_MOESM5_ESM.tiff]

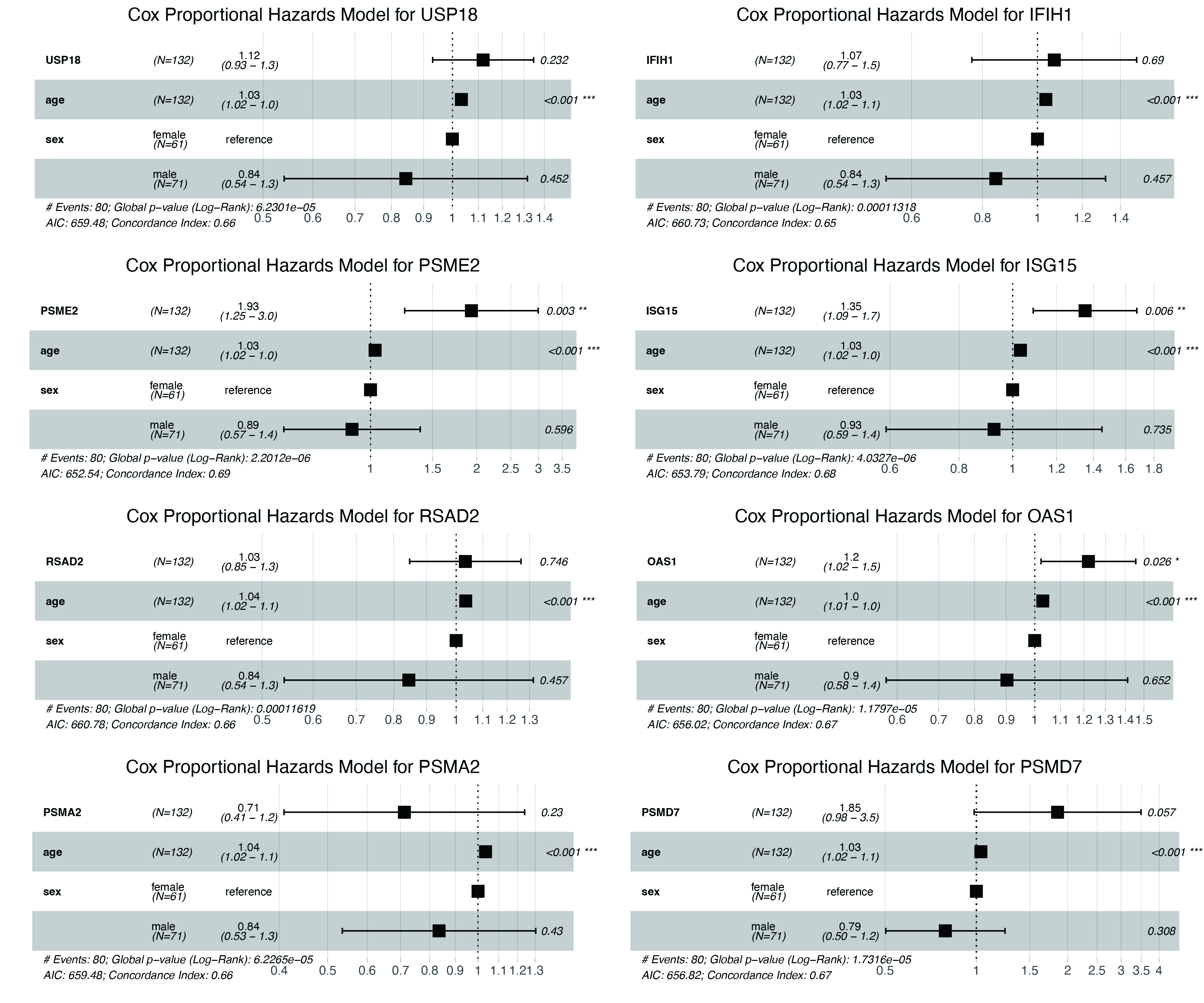

Supplement: Supplementary file 6 — Supplementary Material 6: Figure S6. Core genes significantly associated with prognosis after adjusting for age and gender. Nine core genes were identified through PPI network analysis, with one gene unavailable in TCGA. Multivariable Cox regression analysis was performed using the remaining eight genes. The forest plot in the figure presents the results. [file 12672_2025_2298_MOESM6_ESM.tif]
